# Fractures of the Zygoma, or Zygomatic Arch

**Published:** 1857-11

**Authors:** Frank Hastings Hamilton


### BUFFALO MEDICAL JOURNAL

AND

### MONTHLY REVIEW.

VOL. 13. NOVEMBER, 1857. NO. 6.

### ORIGINAL COMMUNICATIONS.

ART. I.—*Fractures of the Zygoma, or Zygomatic Arch.* By FRANK HASTINGS HAMILTON, M. D.

The zygoma, strictly speaking, is formed in a great measure by the body of the malar bone, and it is broken whenever the malar bone is completely separated through any portion of its body; but I propose to confine my remarks to that portion only which is composed of the two processes, called respectively the zygomatic processes of the malar, and temporal bones.

Duverney related a case in which a young infant having in his mouth the end of a lace spindle, fell forward and thrust the spindle through the mouth from within outward, breaking the zygoma in the same direction, and leaving the fragments salient outward.*

I know of no other example in which the fragments have been thrust outward. A reference to my experiments upon the naked skull will, however,

* Bulletin de la Societé Anatomique, pp. 138, 1810.

show that the zygoma may be broken in the same direction, by any force which shall fracture the superior maxilla, and depress the anterior margin of the malar bone. In my experiments this has happened three times, and always at the same point, viz., a little beyond the middle of the zygoma, near where the suture which joins the two processes terminates below. The fractures were always transverse, and not in the line of the suture. They were therefore fractures of that portion of the zygoma which belongs to the temporal bone.

I suspect, also, that to this class of cases belongs the example related by Dupuytren. in which the patient having died on the fifth day, from the effects of the cerebral concussion, the autopsy disclosed "a fracture through the zygomatic arch; and that part of the superior maxillary bone which constitutes the antrum was driven in."*

In another case mentioned by Dupuytren, produced by a direct blow, the fracture was compound and comminuted, and although the fragments were raised easily by an elevator, suppuration ensued beneath, and the matter was discharged within the mouth.†

Tavignot reports a case of fracture of this arch which was not discovered until after death, the fragments not being at all displaced.‡

Dr. John Boardman, one of the surgeons to the Buffalo Hospital of the Sisters of Charity, informs me that he has lately met with a fracture of the zygoma in a man about 30 years of age, occasioned by a blow from a cricket ball. Dr. Boardman saw him on the fourth day, and ascertained that immediately on the receipt of the injury he felt slightly stunned, and that he soon recovered from this, but was unable to open his mouth except by pulling it open with his hand; neither could he close it except in the same manner. This immobility of the jaw continued several days with only very slight improvement; at the end of five weeks, however, when last seen, the mobility was nearly, but not quite restored. The depression, a little in front of the centre of the zygoma, was discovered by the patient himself immediately after the receipt of the injury, and he says he tried at once to ascertain whether he could not push it back by moving the jaw. He was unable to make any impression upon it by this manœuvre. The depression still remains, but it is not so distinct as it was when first seen.

---

* Injuries and Diseases of Bones. By Baron Dupuytren. Syd. edition. London. 1847. p. 336.

† Op. cit., p. 335,

‡ Bulletins de la Soc. Anat. 1810. p. 138

*Symptoms.* An irregular projection or depression of the fragments are the only signs which can be relied upon to indicate the existence of this accident; and this must often be concealed by the swelling, which follows so rapidly wherever the integuments are severely bruised over a superficial bone. This displacement can scarcely occur in but two directions, either outward or inward; since the attachments of the temporal aponeurosis above, and of the masseter muscle below, must effectively prevent its descent or ascent.

Neither motion nor crepitus will often be present. In some few cases the difficulty in opening or shutting the mouth, occasioned by the projection of the fragments toward or into the tendon of the temporal muscle, may assist in the diagnosis.

*Prognosis.* If the fracture has been produced indirectly by a depression of the malar bone, the prognosis must depend upon the amount of injury done to the other bones of the face; in itself, the fracture of the zygoma cannot be a matter of any moment. The same remark might apply also to any fracture of the zygoma in which the angles were salient outward. If, on the contrary, the angle is salient inward, the fracture having been produced by a blow inflicted directly upon the zygomatic arch, from without, or by a blow upon the outer portion of the malar bone, it may, perhaps, occasion some embarrassment to the action of the temporal muscle.

If the force which produces the fracture has acted more upon the temporal portion of the arch, near where the process arises from the temporal bone, it may be accompanied with a fracture of the skull, and with serious cerebral lesions, as in one of the cases already alluded to as having been noticed by Dupuytren.

The abscess which followed in the case of the compound, comminuted fracture, quoted from the same author, indicates the danger of this complication; but it must be noticed that its evacuation resulted in a rapid cure, and that no deformity or difficulty in moving the jaw remained.

*Treatment.* A fracture, accompanied with an outward displacement, and occasioned by a depression of the malar bone, will be adjusted by a restoration of the malar bone in the manner already described, when speaking of fractures of the superior maxillary, &c. If the fragments are displaced outward, in consequence of a direct blow from within, then they may be replaced by pressing upon the projecting angle. In this way Duverney easily reduced the bones, in the case which I have cited.

When the fragments, in consequence of a direct blow from without, have been driven inward, and, as a consequence, serious embarrassment to the motions of the temporal muscle ensues, an attempt ought to be made at once to replace them; if, however, no impediment to the action of the muscle exists, it is scarcely necessary to say that no surgical interference will be required. It is quite probable, indeed, that a slight amount of embarrassment may be the result of the direct injury to the muscle inflicted by the blow, without reference to the displacement of the bone, and that a few days will suffice to remedy this evil entirely; and, moreover, experience teaches that in the case of a fracture in other bones, where the fragments actually penetrate the muscles and remain thus displaced, the points are gradually absorbed, and rounded so that after a time they constitute no impediment to the action of the muscles. It is proper to infer that the same thing will occur here. The surgeon may be reminded, also, that it is not the muscle but only its tendon which is liable to be penetrated, and that even this is usually protected somewhat by a plate of soft adipose tissue.

If to these considerations we add the difficulties which we shall be likely to encounter in the reduction, we shall expect to find but few cases in which a resort to surgical interference will be necessary.

Duverney says that he restored a fracture of this arch, accompanied with depression, by pressing against the zygoma from within the mouth; but an examination of the interior of the buccal cavity will convince us that this is impossible when the fracture is at any point near the middle of the zygoma, and that it can be only when the fracture is at or near the junction of the zygoma with the body of the malar bone that any effective pressure can be made from this direction. In such a case, we may, perhaps, lift the portion of the zygoma remaining attached to the malar bone, by the same means which have already been suggested for lifting the bone itself.

If the bone is driven toward the tendon of the temporal muscle at or near its centre, as happens almost always, then if its restoration becomes necessary, it can be accomplished only by approaching the bone from without.

Dupuytren found an external wound through which, by the aid of a levator, he easily restored the fragments to place.

M. Ferrier, however, of the Hospital of Arles, in a case brought before him, made an incision through the integuments down to the bone, and then attempted to slide underneath the small extremity of a spatula; but the aponeurosis would not yield, and he was obliged to cut it also. He was now able to lift the fragments easily. The wound healed rapidly and the patient was dismissed without any deformity.*

* Bulletin des Sciences Méd. Tom. 10, p. 160.

